# Hepatitis C Elimination in Egypt: Story of Success

**DOI:** 10.3390/pathogens13080681

**Published:** 2024-08-12

**Authors:** Asmaa Gomaa, Mohamed Gomaa, Naglaa Allam, Imam Waked

**Affiliations:** 1 Hepatology and Gastroenterology Department, National Liver Institute, Menofia University, Shebin El-Kom 32511, Egypt; aibrahim@liver-eg.org (A.G.); naglaaallam@yahoo.com (N.A.); 2 Faculty of Medicine and Health Sciences, University of Sherbrooke, Sherbrooke, QC J1H 5N4, Canada; mohamed.gomaa@usherbrooke.ca

**Keywords:** HCV, Egypt, DAA, NCCVH

## Abstract

Egypt has long been overwhelmed by the hepatitis C virus (HCV) infection, and it used to be the country with the world’s highest prevalence rates. The disease had been a significant public health problem, affecting millions of Egyptians and posing severe economic and social challenges. By the early 2000s, it was estimated that around 10% of the Egyptian population was infected with HCV. However, in recent years, with the availability of direct-acting antiviral therapies, the country has made enormous steps in combating this public health threat. The combination of innovative health strategies and political will enabled Egypt to establish a successful model of care for HCV management and to be the first country to eliminate hepatitis C, setting a model for the rest of the world. In 2023, Egypt became the first country to fulfill the World Health Organization’s set programmatic criteria of reduction of hepatitis C incidence and mortalities to levels close to elimination of disease or achieve the “gold tier” status on the path to disease elimination.

## 1. Background

Liver disease has been a major health problem in Egypt for millennia. With the discovery of the hepatitis C virus (HCV) and the availability of a serological test, it became apparent that HCV was causing most liver disease in the country, with prevalence levels the highest in the world [1]. Local epidemiological studies in 2008 and 2015 revealed high hepatitis C virus (HCV) prevalence rates, with national estimates ranging from 10% to 15% of the population being seropositive for HCV antibody and 10% chronically infected (about 6 million people viremic) and accounting for 7.6% of the country’s mortality. In rural Egypt, the prevalence of HCV was higher, reaching 24%, which is 10–20 fold higher than in the United States [2,3]. The HCV incidence rates were estimated at 2.4 per 1000 person-years, with nearly 165,000 new infections annually in 2010. Until 2015, Egypt had the highest prevalence rate of hepatitis C in the world. According to World Bank estimates, more than 1 in 5 Egyptians aged 50–59 had hepatitis C, leading to a decline in productivity by 7.5% amongst infected people [4]. Genotype 4 (G4) represented more than 90% of HCV isolates from Egyptian patients [5].

The main cause for the high prevalence of HCV infection in Egypt was the parenteral anti-schistosomal therapy, which was used to treat schistosomiasis from the 1950s to the early 1980s in national mass therapy campaigns, mostly with poorly sterilized reusable glass syringes, thus spreading the infection to a huge cohort [6]. Although this was replaced by oral schistosomal therapy in the 1980s, poor infection control, equipment sterilization, and blood safety procedures in the healthcare setting at the time led to the continued spreading of the disease.

The Egyptian government recognized the urgent need to address the HCV epidemic, and in 2006, the Ministry of Health and Population (MoH) established the National Committee for the Control of Viral Hepatitis (NCCVH), tasked with developing and implementing a national plan to combat hepatitis C [7]. The NCCVH board is formed from volunteer experts and stakeholders from various fields, including hepatologists/gastroenterologists, infectious disease specialists and epidemiologists, pharmaceuticals, and policy-makers. They set the necessary plans for the management of viral hepatitis, which aimed to reduce the prevalence of hepatitis, improve treatment outcomes, and raise public awareness. They started to collect accurate epidemiological data about HCV viremia and transmission and included HCV serology and viremia in the 2008 and 2015 Egypt Demographic and Health Surveys (DHS) [8,9]. The NCCVH set treatment guidelines for optimal management and recommendations for ideal prevention and infection control that were adopted by the MoH and insurers. With only 30% of the population covered by the national health insurance and 5% by private insurers, and with 65% paying out-of-pocket, the government agreed to treat all uninsured HCV patients at the expense of the state so that all patients will have access to diagnostics and treatment regardless of their financial ability. 

## 2. The NCCVH Program in Interferon Treatment Era

Before 2014, dual therapy with pegylated interferon (PEG) and ribavirin (RBV) for 48 weeks had been the standard of care in patients with HCV. Sustained virologic response (SVR) rates for patients treated with PEG-RBV therapy given for 24 weeks or 48 weeks ranged from 40% to 50% [10], depending on HCV genotype, patient’s fibrosis scores [10], and adherence to the treatment regimen [11]. Baseline viral load [12], IL-28B genotype [13], and insulin resistance [14,15] were the main predictors of response. Real-life data from a worldwide PROPHESYS study showed an SVR rate of 41.5% for patients without bridging fibrosis or cirrhosis; however, it dropped to 27% for those who had bridging fibrosis or cirrhosis [10]. Similar SVR rates were observed in Egyptian patients infected with HCV genotype-4 [16]. 

The NCCVH set up specialized interferon treatment centers across the country, where patients were managed by trained hepatologists and gastroenterologists experienced in interferon therapy and its complications. This started with a single center in 2007, reaching up to 26 centers by 2014. The NCCVH implemented national programs to facilitate access to interferon therapy for those who could not afford the expensive medication. Extensive public health campaigns were launched to raise awareness about HCV, its transmission, and the importance of getting tested and treated. 

Given this very low rate of SVR (27–41%), the NCCVH decided to offer the treatment to patients with biopsy-proven F2-F3 fibrosis only. Patients with no or F1 fibrosis were deferred to be treated when better treatment options became available. Patients with F4 fibrosis, previous treatment failures, aged over 60, or who have high body mass index were excluded from treatment at the time. Approximately 350,000 patients were treated by the Egyptian national program for the treatment of hepatitis C between 2007 and 2014; real-life data from Egypt showed an SVR rate of 45–55% [17].

PEG-RBV therapy was associated with significant side effects, including flu-like symptoms, fatigue, depression, anemia, and other hematological abnormalities. These side effects often led to poor adherence and early discontinuation of therapy. In addition, PEG-RBV had many contraindications, including decompensated cirrhosis [18]. Fibrosis stage determination necessitated liver biopsy, which added another obstacle to treatment.

Between 2007 and 2017, PEG-RBV-based therapy resulted in more than 200,000 IFN treatment failures; 300,000 were postponed due to the absence of fibrosis or presence of cirrhosis, and a few hundred thousand refused to receive interferon because of reported low efficacy and adverse events. This led to a pool of more than 700,000 untreated patients diagnosed with HCV infection waiting eagerly for new antiviral treatment.

### 2.1. First Generation Protease Inhibitors

In 2011, the first directly acting protease inhibitors, boceprevir [19] and telaprevir [20], were approved and were used in combination with PEG-RBV for patients with genotype 1, starting a new era in HCV infection management. This combination increased SVR rates up to 70%; however, they were ineffective for patients infected with HCV G4, and unfavorable adverse effect profiles were reported. The low response rate and the side effects of therapy mandated the search for alternative treatments.

### 2.2. Combination of Direct-Acting Antiviral Drugs with PEG/RBV

The first oral direct-acting antiviral drugs effective for HCV-G4 were simeprevir (SMV), a nucleotide protease inhibitor, and sofosbuvir (SOF), a nucleotide polymerase inhibitor. These agents were well tolerated and approved by the Food and Drug Administration (FDA) in late 2013, followed by daclatasvir (DCV), an NS5A inhibitor, in 2015. SOF is a potent pangenotypic NS5B RNA polymerase inhibitor with a high barrier to resistance that introduced a new era for the treatment of HCV, especially G4, when approved in December 2013. The addition of SOF to PEG-RBV therapy for 12 weeks resulted in an SVR rate of 96% in treatment-naive HCV G4 patients [21]. A study that included patients of Egyptian origin living in the United States infected with HCV G4 showed favorable results from SOF-based treatment (SVR rates of 79 and 59% in treatment-naıve and treatment-experienced patients, respectively) [22]. Other trials conducted in Egypt thereafter established the efficacy of SOF with PEG-RBV or RBV treatment in HCV G4. When SMV was given once a day orally for 12 weeks with PEG-RBV for 24–48 weeks, it improved the SVR in HCV-G4 treatment-naive patients to 83% and previous treatment-experienced patients to 86% in prior relapsers and 40% in prior non-responders [23].

The addition of an oral daily dose of 20 mg of DCV to a 24week course of PEG-RBV improved SVR rates in HCV-4 patients to 67% and to 100% with a 60 mg dose compared to 50% with standard 48-week PEG-RBV treatment [24]. Thus, by the end of 2014, the NCCVH introduced SOF-based treatment, followed by SMV and DCV treatment regimens, to the national treatment program in Egypt [25]. 

### 2.3. IFN-Free Direct-Acting Antiviral Drugs

Additional DAA medications were approved, and IFN-free treatments became available for all HCV genotypes. Several well-tolerated regimens were approved to treat patients with various HCV genotypes, stages of liver disease, and comorbidities. For treatment of HCV G4 patients, paritaprevir 150 mg, ritonavir 100 mg, and ombitasvir 25 mg were used. Paritaprevir is an inhibitor of the NS3/4A serine protease, ombitasvir is an NS5A inhibitor with pangenotypic antiviral activity, and ritonavir is used as a CYP3A4 and CYP2D6 inhibitor, which is present to decrease the breakdown of paritaprevir allowing the use of a lower dose [26,27]. 

The NCCVH’s SOF price negotiations with Gilead Sciences resulted in a drastic 99% discount from the US market price of USD 28,000 to USD 300 for a bottle of 28 pills sufficient for a 4-week supply. This enabled the development of the largest treatment program in the world. Negotiations with the other DAA manufacturers led to comparable price reduction; the price of a 4-week supply of SMV and DCV was reduced to USD 250 each, paritaprevir-ombitasvir to USD 300, and sofosbuvir/ledipasvir (SOF-LDV) to USD 400 [28]. 

In 2016, the Egyptian government encouraged local pharmaceutical companies to produce generic DAAs (SOF, DCV, and SOF-LDV) at a fraction of the international cost. The cost of a 12-week treatment with SOF-DCV for the National program dropped from USD 900 to less than USD 300 in 2016 and later to less than USD 100 in 2018. The use of generic DAAs ensured the treatment’s accessibility and affordability, leading to a significant reduction in the disease burden.

## 3. The DAA Treatment Program in the NCCVH

The National DAA treatment program for hepatitis C in Egypt is a public health initiative that has gathered international recognition for its success in combating a major health crisis. The National DAA treatment program was a collaborative effort between the Egyptian government, local pharmaceutical companies, and international health organizations. The program, launched in October 2014, aimed to provide widespread access to effective antiviral medications directing at controlling hepatitis C in Egypt [29]. IFN-based therapy left more than 1,000,000 HCV patients waiting eagerly for the new antiviral treatment. These included more than 200,000 previous IFN treatment failures, patients without significant fibrosis (F0-F1 on biopsy) who were deferred for later treatment, patients with F4 fibrosis, decompensated cirrhosis, and severe obesity who were not considered for IFN treatment, and many who refused to have a liver biopsy or to undergo IFN therapy. The challenge of organizing and managing these patients to commence treatment was immense and required innovative approaches.

In 2014, the NCCVH committee developed a comprehensive strategy [2] that included the following:

- Screening and Diagnosis: Implementing widespread screening programs through the initiation of mass screening campaigns across the country, targeting millions of people to detect HCV in high-risk populations, including those over the age of 18 and individuals with high-risk behaviors.
- Public Awareness and Education: Raising public awareness about HCV transmission and prevention. Awareness educational campaigns were launched to inform the public about the disease, its transmission, safe medical practices, the importance of screening, and available treatment options.
- Access to Treatment: Egypt negotiated with pharmaceutical companies to produce generic DAAs, significantly reducing the cost of treatment. The use of generic DAAs secured the treatment’s accessibility and affordability, leading to a significant reduction in the disease burden.
- Healthcare Infrastructure: Enhancing healthcare infrastructure to support diagnosis, treatment, and follow-up. Specialized treatment centers were established across the country and were equipped with the necessary infrastructure and trained healthcare personnel. A robust monitoring and evaluation system was put in place to track the progress of patients and ensure treatment efficacy.

The NCCVH implemented a web-based registration and appointment management system to streamline the process of diagnosing and treating hepatitis C patients. This system played a crucial role in managing the large number of patients requiring treatment, allowing patients to register online with their name, national ID number, and address, to be scheduled for the earliest available appointment at the nearest center to their residence. Patients were assessed for treatment at centers overseen by the NCCVH or the National Health Insurance Organization (HIO). The daily workload and appointments were adjusted based on each center’s capacity. Patients received their appointment confirmations through the web portal and via text message. On the portal’s launch day, 103,000 patients registered for treatment. This number grew to more than 500,000 within the first month and reached approximately 1,500,000 by the end of November 2016.

Patients underwent clinical examinations, biochemical tests and blood counts, viral load testing, and an abdominal ultrasound. In the first three months, treatment was available for only 50,000 patients. During this period, healthcare providers had to select patients for treatment and prioritize treatment for patients with advanced fibrosis or cirrhosis, restricting treatment to those with F2-F4 fibrosis as long as the patients had compensated cirrhosis while others were deferred till the availability of more medications. This fibrosis stage was initially determined using liver stiffness measurement by Fibroscan. However, due to the limited number of Fibroscan machines at that time, FIB-4 was used as a measure to evaluate fibrosis, where patients with FIB-4 values > 3.25 were considered to have cirrhosis, while patients with FIB-4 < 1.45 were considered to not have significant fibrosis and were deferred for later treatment. 

Prior to the introduction of any new brand or generic DAA into the national HCV treatment program, local clinical trials were conducted to verify efficacy in HCV genotype 4 [24,25,27,30,31,32,33,34]. The initial treatment protocol involved a 12-week regimen of SOF-PEG-RBV for treatment-naive patients without cirrhosis, while those who had previously failed PEG-RBV treatment or had cirrhosis were given a 24-week regimen of SOF-RBV. Real-life data showed SVR12 rates were 94% in those who received SOF-PEG-RBV and 78.7% in SOF-RBV regimen [35]. Treatment protocols were continuously updated to incorporate new medications and address administrative challenges. During the first 18 months, several adjustments were made to include more patients, adapt treatment regimens based on emerging results, and integrate new drugs as they became available. Cost considerations were pivotal in decision making, and it was essential to select regimens suited to HCV G4. To improve patient flow, the number of evaluation and treatment centers was expanded to over 150 centers, which were managed by both the NCCVH and the National HIO. By mid-2016, with an increased medication supply and the introduction of locally manufactured generics and more treatment centers, all patients were evaluated and began treatment within a week of registering on the portal.

SOF-based regimens remained the only available DAA in Egypt until March 2015, and over 150,000 patients began treatment. At that time, SMV was registered and became available, leading to the replacement of the 12-week SOF-PEG-RBV regimen with a SOF-SMV combination for patients without cirrhosis. The 24-week SOF-RBV regimen for patients with cirrhosis remained unchanged until DCV was introduced. Once DCV became available, the supply of SMV was reserved for patients with renal impairment, as DCV was much less expensive than SMV.

In 2015, Egypt made significant progress in its battle against hepatitis C by successfully treating over 400,000 patients. The subsequent availability of locally produced generic SOF and DCV in early 2016 ensured a steady and sufficient supply of these essential medications. The reduced cost made it financially feasible to extend treatment to a broader population and enabled the healthcare system to meet the high demand for HCV treatment. With easy accessibility to medications, the need to prioritize patients based on the severity of fibrosis or cirrhosis was eliminated, allowing treatment of all HCV-infected individuals, irrespective of the stage of fibrosis. This inclusive approach significantly increased the number of patients receiving treatment and improved overall public health outcomes. 

Treatment-naive patients without cirrhosis were considered as “easy to treat” and were treated with SOF-DCV for 12 weeks, while previous treatment failures or patients with FIB-4 > 3.25 were considered as “difficult to treat” and were treated with a combination of SOF-DCV-RBV for 12 weeks (or SOF-DCV for 24 weeks if they had decompensated cirrhosis or were RBV-intolerant to enhance efficacy). Nearly all patients received treatment regimens consisting of SOF combined with other DAAs. Only a small minority, approximately 3%, were treated with paritaprevir-ritonavir-ombitasvir (PrO), primarily reserved for patients with renal impairment. Real-world SVR rates for SOF-containing regimens were 94% with SOF-PEG-RBV, 83% with SOF-RBV, 97% with SOF-SMV, and 98.5% with SOF-DCV [34,36]. In cases of DAA treatment failure, patients were retreated using SOF-SMV-DCV or SOF-PrO regimens with RBV unless they were RBV-intolerant and had anemia or heart problems.

With the very high SVR rate and low cost of these DAA combinations, more recent DAAs were not introduced to the national treatment program. The glecaprevir/pibrentasvir combination was FDA-approved in 2017 when the Egyptian program was mainly using locally manufactured generics at a cost of USD 100 for a treatment course. The manufacturer did not see the country as an interesting market, and the combination was not introduced to the national program. On the other hand, SOF-velpatasvir (VEL) was evaluated in a small retreatment clinical trial in Egypt [37]. It was registered as a locally manufactured generic, was not introduced to the national program, and was available only in private practice. The triple combination SOF-VEL-voxileprevir is used for the retreatment of treatment failures and relapsers in the national program.

By 2018, over 2 million patients had started treatment, having been previously diagnosed with hepatitis C. However, the monthly registration of new patients dropped to less than 10,000, which is significantly below the required number to meet the elimination target of treating at least 350,000 patients annually [38].

## 4. National Screening Program

The success of the HCV national treatment strategy 2014–2018 encouraged the government to shift its strategy from HCV control to elimination. At the time, it was estimated that between two and three million patients infected with HCV remained undiagnosed and unaware of their disease. Since early diagnosis enables timely effective treatment which can prevent the progression of liver disease and reduce transmission rates, and in view of the availability of large supply of SOF and the lower prices reached in early 2018 (less than USD 100 for a 12-week supply of SOF-DCV), the NCCVH implemented a comprehensive national screening program to screen the entire adult population (a target group of 62.5 million) and teenage school-children in middle and high schools (a target group of 12 million) to identify and treat undiagnosed HCV infections [39]. Preparation for this screening program took from July to October 2018 (preparation and training of the medical teams, negotiations with suppliers, and procurement).

All persons above the age of 18 were invited to participate in the screening program. WHO pre-qualified rapid test for HCV antibody with immediate results at a low cost of USD 0.56 per test was used for initial screening to identify HCV antibodies in a blood-drop. Results were immediately electronically registered to a central database using computers or handheld devices via cellular networks. Patients who tested positive in the initial screening were immediately registered for an appointment for further evaluation at the closest evaluation and treatment center. This included liver tests, viral load tests (PCR for HCV-RNA at a negotiated low cost of less than USD 5 per test), and an ultrasound examination. The linkage to care was streamlined to ensure that patients received timely and appropriate treatment. Nearly 50 million people were screened for HCV antibodies, 2.4 million seropositive people were identified, and 1.6 million viremic patients were identified and treated. Viremic patients received 12 weeks SOF-DCV +/- RBV, with SVR rates higher than 98% over a period of 7 months.

Screening for the teenage population took place at school, provided parents signed a consent form agreeing to their child being tested for HCV and treated if infected. To prevent stigmatization, neither students nor school staff were informed of the results, which were instead mailed to parents. Positive children were then scheduled for evaluation and treatment at health insurance clinics away from their schools. Of the 7 million teenagers who participated in the screening, 20,000 (0.3%) were identified as seropositive and subsequently evaluated. The treated children achieved a 100% SVR rate. This initiative was the first teenage screening and treatment program for hepatitis C in the world [40].

## 5. Outcome of the National Screening and Treatment Program

**Figure 1:**
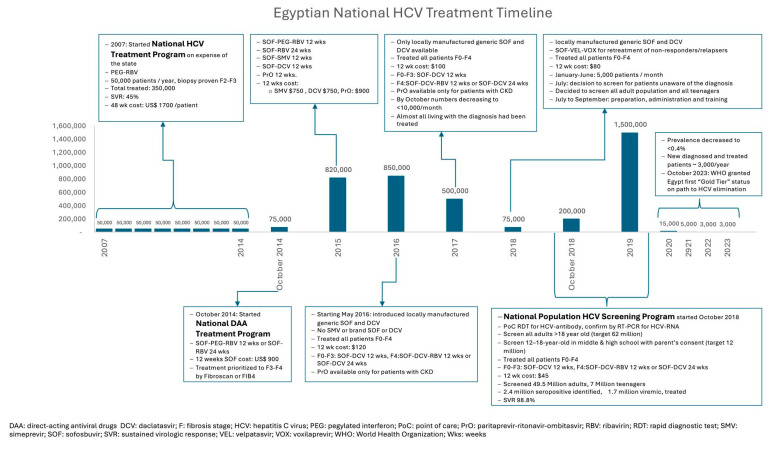
Timeline and different therapies and patient inclusion of the Egyptian HCV treatment programs, 2007–2023.

Figure 1 outlines the timeline, different therapies, and patient inclusion of the Egyptian HCV treatment programs, from initiating the national program of PEG-RBV therapy in 2007 until achieving the “gold-tier” of near elimination of the disease in 2023. The national DAA treatment program that started in 2014 and the national screening and treatment program of 2018–2019 resulted in the treatment of more than 4 million HCV patients with SOF-based treatment, achieving SVR rates above 95% [2]. HCV prevalence is estimated to have decreased from 6% of the population in 2015 to less than 0.5% of the population in 2021 (decrease from 5 million patients to less than 400,000 patients). Egypt achieved all the WHO elimination targets from the diagnosis of more than 90% of its HCV-infected population, offering treatment for more than 90% of them and a cure for more than 95% of them [41]. 

In a modeling study, the total number of viremic cases is expected to decrease in 2030 by 86%, resulting in the prevention of 250,000 new infections between 2020 and 2030, and the program would result in averting more than 1 million disability-adjusted life years (DALYs), preventing close to 250,000 HCV-related mortalities and more than 150,000 new cases of hepatocellular carcinoma (HCC) [42,43].

This recent success of the national treatment programs in markedly reducing the prevalence of viremic infection, coupled with blood safety standards reaching 100% and improved hospital infection control in all public hospitals, are leading to a marked reduction of HCV transmission and near elimination of the disease [44,45,46]. The incidence of HCV infection in the high-prevalence Nile Delta region was estimated at 6.1 per 1000 person-years in 2005 [47] and decreased to 2.4 per 1000 person-years in 2010 [48]. A study evaluating the impact of the treatment program before establishing the national screening campaign reported the incidence rate to be 0.37 per 1000 person-years in the same area, representing an 84.6% reduction [49]. The national screening and treatment campaign in 2018–2019 is projected to have further reduced the incidence to 0.07 per 1000 person-years, or around 7000 new cases per year, down from an estimated 150,000 new cases annually before the implementation of these programs, representing a reduction in the incidence of over 95%. 

To verify the effectiveness of the National Screening and Treatment program, the Ministry of Health, in 2022, conducted a survey that included a nationally representative sample of 20,881 subjects where they found that 92 (0.4%) were HCV-infected and that the prevalence of HCV infection had decreased by 93% compared to 2015 [50]. Similar surveys are planned be repeated at regular intervals.

The economic impact of the treatment program is remarkable despite the significant costs of the treatment program (USD 350 million) between 2014 and 2018 and the screening and treatment campaign (USD 207 million) in 2018–2019, the total economic gain considering both direct and indirect costs is estimated to exceed USD 7 billion from 2020 to 2030 [42].

The WHO established a “gold tier” target, aiming to diagnose at least 80% of people with hepatitis C and provide treatment to at least 70% of those diagnosed. Egypt diagnosed 87% of the people living with hepatitis C and provided 93% of them with curative treatments [41].

On 9 October 2023, the WHO praised Egypt for its remarkable progress toward eliminating hepatitis C and announced that Egypt had become the first country to achieve the “gold tier” status on the path to the elimination of hepatitis C as per WHO criteria, indicating that Egypt has met the programmatic requirements necessary to significantly reduce new hepatitis C infections and its related mortality [51,52]. Table 1 summarizes what we consider the main success factors for the HCV programs, mainly the national screening and treatment program in Egypt. These can be considered.

In conclusion, Egypt has successfully treated over four million patients with chronic HCV infection and became the first country to achieve WHO validation on the path to elimination of hepatitis C. Some of the factors that were essential in making this program affordable and successful are outlined in Table 1. What was also important was the resilience in decision making and program change according to the availability of better therapies, lower cost, or emergent problems. The dependence of the program on locally produced generic SOF-DCV therapy, despite the availability of newer DAAs and international guideline recommendations, was based on locally generated real-life efficacy and safety data and it being the most affordable regimen. The prevalence of viremic infection in the country has dropped from 7% (5.5 million patients) in 2015 to an estimated 0.4% in 2021. The treatment program has a significant economic impact. Egypt stands on the edge of potentially eradicating HCV, setting a model for the rest of the world.

## Figures and Tables

**Table 1:** Lessons learned from the Egyptian National HCV elimination program (why the program was successful and what can be replicated elsewhere).

Success Factors and Lessons Learned from the Egyptian HCV Elimination Program
Societal pressure is essential to drive policy-makers to adopt a national treatment program.
Continuous political will and support are crucial for initiating and maintaining a successful program.
Allocation of sufficient resources to initiate and maintain the full program
An empowered central decision body to set guidelines and recommendations
Management guidelines are simple, allowing task-shifting to non-specialists.
Guidelines are adaptable, reflecting the most effective regimens, availability, and cost.
Mass procurement through a single negotiating body ensures cheap prices for investigations and treatment
All services are free to patients (diagnostics, treatment, follow-up); this is a key factor driving compliance and program success.
Treat all patients regardless of fibrosis stage, using simple biomarkers of fibrosis to decide on follow-up.
Population screening feasible, community engagement and mobilization are possible.
Immediate effective linkage to care and treatment is essential.
Limited number of visits (one screening visit, one evaluation visit, one visit for full treatment dispensing, one visit to assess SVR)
Inclusive strategies for PWID, PLHIV, and people who are incarcerated
Huge media and ad campaign

## Data Availability

The data for the National DAA Treatment program and the National Screening Program (includes data for more than 2.5 million patients) are available in the database of the National Committee for the Control of Viral Hepatitis in Egypt. The raw data are confidential and cannot be released. Results of data analysis have been previously analyzed and published and are available in the literature and can be provided by the authors upon request.
